# Magnetic resonance spectroscopy in hospitalised older people shows age and delirium-specific metabolic changes

**DOI:** 10.1093/ageing/afag013

**Published:** 2026-02-01

**Authors:** Daniel Richardson, Aisha Mahmood, Lauren Binnie, Uzma Khan, Philip Rich, Daniel H J Davis, Atticus H Hainsworth, Franklyn A Howe, Jeremy D Isaacs

**Affiliations:** Emergency Medicine, King’s College Hospital NHS Foundation Trust, London, England, UK; Neuroscience & Cell Biology Research Institute, School of Health & Medical Sciences, City St George's, University of London, London, England, UK; Neuroscience & Cell Biology Research Institute, School of Health & Medical Sciences, City St George's, University of London, London, England, UK; Department of Neurology, Atkinson Morley Regional Neurosciences Centre, St George’s University Hospitals NHS Foundation Trust, London, England, UK; St George's Vaccine Institute, School of Health & Medical Sciences, City St George’s, University of London, London, England, UK; Department of Neurology, Atkinson Morley Regional Neurosciences Centre, St George’s University Hospitals NHS Foundation Trust, London, England, UK; MRC Unit for Lifelong Health and Ageing, University College London, 33 Bedford Place, London WC1B 5JU, England, UK; Neuroscience & Cell Biology Research Institute, School of Health & Medical Sciences, City St George's, University of London, London, England, UK; Department of Neurology, Atkinson Morley Regional Neurosciences Centre, St George’s University Hospitals NHS Foundation Trust, London, England, UK; Neuroscience & Cell Biology Research Institute, School of Health & Medical Sciences, City St George's, University of London, London, England, UK; Neuroscience & Cell Biology Research Institute, School of Health & Medical Sciences, City St George's, University of London, London, England, UK; Department of Neurology, Atkinson Morley Regional Neurosciences Centre, St George’s University Hospitals NHS Foundation Trust, London, England, UK

**Keywords:** delirium, cognition disorders, humans, frail, dementia, magnetic resonance spectroscopy, older people

## Abstract

**Background:**

Delirium is common in hospitalised older people and is associated with a poor prognosis. It remains poorly characterised at a molecular level. We studied the metabolic signature of delirium using ^1^H-Magnetic Resonance Spectroscopy (MRS) in a prospective case-control study.

**Methods:**

Medical inpatients aged ≥65 with and without delirium (DSM-5) were recruited and assessed for illness severity, frailty and prior cognitive decline. Metabolite concentrations in parietal white matter were obtained using MRS with diffusion MRI used to assess structural changes via the ADC.

**Results:**

Out of 38 participants, 25 completed the MRS protocol (13 males and 12 females, mean age 80.5, SD = 6.47). Patients with delirium (*n* = 13) had greater pre-admission frailty than those without (*n* = 12) (median Clinical Frailty Scale 5 vs. 4.5; *P* = .049). There were no significant differences in age, sex, measures of MRS quality, atrophy and white matter disease. In a General Linear Model using the MRS voxel ADC to account for white matter lesion effects, glutamate was higher in delirium patients (*P* = 0.024). There were no other between-group differences in metabolite concentrations. For patients with and without delirium combined, glutamine increased with age and decreased with cortical atrophy, whilst Myo-inositol decreased with age and increased with median ADC.

**Conclusions:**

Our results suggest that delirium is characterised by elevated brain glutamate concentration. This could cause excitotoxic brain injury and contribute to post-delirium cognitive decline and is a potentially modifiable process that merits further investigation.

## Key points

- Cerebral glutamate (Glu) concentrations were ~15% higher in older adults with delirium compared to age- and illness-matched controls. This suggests a possible role for glutamatergic dysregulation in delirium pathophysiology.
- Glu excitotoxicity may be a modifiable mechanism linking delirium with long-term cognitive decline, driven by impaired astrocytic Glu uptake and neuroinflammation in vulnerable ageing brains.
- Myo-inositol was associated with white matter damage and decreased with age, possibly reflecting glial dysfunction in later life, independent of delirium status.
- This is the first MRS study in a typical frail older hospitalised population with delirium, providing evidence of distinct metabolic profiles in this population.

## Introduction

Delirium is an acute disorder of consciousness characterised by decline in cognitive function, perceptual disturbances, altered behaviour and physical impairment [1]. Delirium is a common complication affecting older hospitalised patients, with an estimated prevalence of 18%–30% in medical inpatients aged over 65. Delirium prevalence rises steeply with age—affecting nearly one in five aged 65–74, one-third of 74–89 year olds, and half of all patients aged 90 or older [2, 3].

Delirium is associated with multiple adverse outcomes, including an 8 to 12-fold increase in dementia incidence [4–6]. Whilst previous authors have identified the importance of preventing or intervening in delirium to prevent or postpone the onset of dementia, no established therapeutic targets currently exist [7].

An emerging body of evidence demonstrates that delirium is associated with increased levels of neurofilament light (a marker of neuronal injury) and glial fibrillary acidic protein (GFAP) (a marker of astroglial activation) [8] but how neurons are injured in delirium remains unknown; post-delirium cognitive decline is not fully accounted for by dementia-related neuropathology [7, 9, 10]. Potential pathways include neurotransmitter or metabolic dysfunction.

Dysregulated neurotransmitter systems have been implicated in the aetiology of several neurological disorders [11]. L-glutamate (Glu), the principal excitatory neurotransmitter in the human brain, has been linked to a variety of acute and chronic brain diseases and forms a final common pathway to cellular injury [12–14]. Glu concentrations are generally reduced in neurodegenerative disorders [15] but elevated in acute brain injuries; levels rise in proportion to the degree of infarction in stroke, whilst greater levels in traumatic brain injury predict poorer prognosis [16, 17].

Evidence from animal models of delirium suggests that cerebral Glu or the total pool of glutamine (Gln) and Glu (Glx) is increased and correlates with acute cognitive deficits [18–20]. To date, clinical studies of Glu in delirium have been sparse. Glu concentration in cerebrospinal fluid (CSF) taken from patients prior to surgery for hip fracture was higher in those that developed post-operative delirium compared those who did not [21]. One study of delirium following bone marrow transplantation (BMT) did not find altered brain Glx levels [22]. We hypothesised that concentrations of Glu in the brain are elevated in delirium and thereby contribute to post-delirium cognitive decline.

Acquiring *in vivo* data is a limiting step in patients with delirium who are often frail and have disturbances in arousal and behaviour. 1H-Magnetic Resonance Spectroscopy (MRS) offers a non-invasive method of quantifying concentrations of small molecules in brain tissue [23, 24].

We applied MRS to a typical sample of older hospitalised patients with and without delirium. We ascertained concentrations of Glu and other metabolites in cerebral white matter and investigated whether these were affected by common confounders in this population; age, white matter disease, atrophy, and neurological comorbidity.

## Methods

Ethical approval for this study was obtained from the Wales Research Ethics Committee 6 (18/WA/0063).

Between July 2018 and June 2021, inpatients over 65 years old with and without delirium were recruited from medical wards at a single tertiary centre (St George’s Hospital, London, UK). This was achieved through the research team screening patients on medical wards and being alerted to potentially suitable participants by ward-based medical teams and specialist delirium and dementia nurses. Patient recruitment was generally undertaken on the afternoon of the day prior to the availability of MRI scanning slots to facilitate imaging within 24 hours of assessment.

Patients were included in the delirium group if they met DSM-5 criteria for delirium. ‘No delirium’ patients were selected based on having neither delirium according to DSM-5 nor subsyndromal delirium based on single clinical assessment by an experienced cognitive neurologist (J.D.I.). Patients were excluded if MRI was contraindicated, or if they had an unstable medical condition precluding safe transfer to the MRI scanner or brain pathology likely to severely interfere with MRS signal (such as brain tumour, encephalitis or acute large vessel stroke). No medications were used to assist compliance with MRI.

Assent for participation was provided on behalf of patients without capacity via next of kin or a senior healthcare professional responsible for the patient’s care and not involved in the research project. As this was a pilot project, no sample size calculation was performed.

### Clinical assessment

Presence of delirium according to DSM-5 criteria and its severity on the Observational Scale of Level of Arousal (OSLA) [25] and Memorial Delirium Assessment Scale (MDAS) [26] were assessed on a single occasion. The following clinical assessments were undertaken; APACHE-II (without arterial blood gas ascertainment) as a measure of physical illness severity, [27] Clinical Frailty Scale (CFS) based on the patient’s status immediately prior to the acute medical illness causing admission, [28] and the short IQCODE to screen for pre-admission cognitive decline [29]. Patient medical records were also reviewed for existing medical conditions such as dementia. Participants were regarded as having dementia only if they had a confirmed pre-admission diagnosis. Prior to MRI (if imaging not performed immediately after assessment) the participants clinical status, and consent and suitability for scanning were reassessed.

### MR imaging and spectroscopy

**Figure 1:**
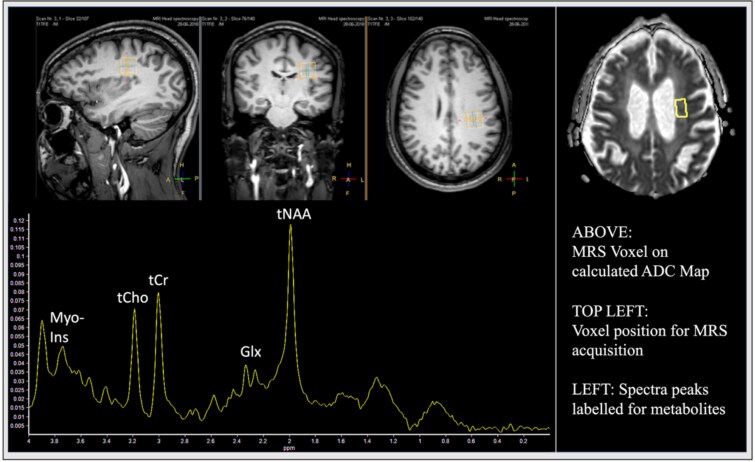
Example 1H-MRS voxel placement and ADC map. Upper row: T1-weighted images indicating voxel position for MRS acquisition in PWM. Top right: ADC image demonstrating voxel placement. Median ADC was calculated from all image slices within the MRS voxel. Lower row: Spectra peaks labelled for metabolites.

A 3T MRI system was used to acquire structural brain imaging and quantitative MRS data (see Appendix S1 in the supplementary methods for details). To evaluate brain structure and identify confounding neuropathology we used: High resolution 3D T1-weighted (T1w), axial Fluid Attenuated Inversion Recovery (FLAIR), diffusion weighted imaging (DWI), susceptibility weighted imaging. Quantitative MRS was acquired from parietal white matter (PWM) (Figure 1) with voxel dimensions chosen to maximise white matter volume whilst excluding signal from CSF and grey matter. PWM was chosen based upon literature indicating widespread atrophy and impairment to white matter integrity in parietal, frontal and temporal regions in delirium, [30] and our experience suggesting it offers a pragmatic choice for selecting a large enough MRS voxel for high signal-to-noise ratio (SNR) spectra in the presence of atrophy [31]. We utilised support from relatives, a skilled and tactful radiographer team and pragmatic prioritisation of MR sequences, including focusing on one brain region to minimise acquisition time, to safely perform MRI/MRS in patients likely to find MRI scanning difficult to tolerate.

T1w and FLAIR images were used for anatomical placement and to minimise contributions from White Matter Hyperintensities (which represent areas of small vessel disease associated with ageing, hypertension, and other vascular risk factors) (Figure 1). This was especially relevant in patients with extensive small vessel disease [32, 33]. MRS data underwent quality control assessment [34] prior to metabolite quantitation (see Appendix S1 in the supplementary methods for details) with results presented as institutional units (IU) for:

- Glu, Gln and their sum (Glx)
- Myo-inositol (mI), found in glial cells and related to glial activation and inflammation
- N-Acetyl aspartate + N-acetyl aspartate glutamate (tNAA), a marker of viable neuronal cells
- Glycerophosphocholine + Phosphocholine + Choline (tCho), a marker related cellular membrane turnover
- Creatine + Phosophocreatine (tCr), the sum of which is fairly constant in healthy tissue with normal energy metabolism, but changes in pathology.

The tissue apparent diffusion coefficient (ADC) within the MRS voxel was used as a covariate in group comparisons to account for variations in cell density and water content (see Appendix S1 in the supplementary methods for details).

Atrophy and global white matter hyperintensity were graded using Global Cortical Atrophy (GCA) scale [35] and Fazekas [36] scores respectively, with assessments performed by a Consultant Neuroradiologist (P.R.) who was blinded to group. GCA grades 13 brain regions on a scale of 0–3 with higher scores indicating greater degrees of atrophy; the maximum possible score is 39. The modified Fazekas scale grades lesions seen on FLAIR/T2 in either deep or periventricular white matter from 0–3; 0 = no lesions, 1 = focal changes or thin lining changes in the periventricular white matter, 2 = small areas of confluence or halo patterning around the ventricles, 3 = large confluent white matter lesions. In our sample no participants had a Fazekas score of 0.

### Statistical analysis

Statistical analysis was undertaken using SPSS 29 and Prism (version 10), a significance level of *P* < .05 was used. Normality tests (Kolmogorov–Smirnov and Shapiro–Wilk) across all data demonstrated significant non-normality for the following parameters: OSLA, APACHE-II, IQCODE, Fazekas score. Therefore Mann–Whitney was used to assess group differences in clinical scores.

The Full-Width Half Maximum (FWHM) and SNR (which depends on MRS voxel size) were included as covariates, as variation in these parameters can produce systematic shifts in metabolite levels as determined by peak fitting algorithms [37]. The Independent-Samples Jonckheere–Terpstra Test for Ordered Alternatives (ISJT) was used to assess whether there was a statistically significant trend for variables to change with Fazekas score. Linear regression was used to provide visual representation of metabolite levels as a function of multiple variables.

### Additional analysis

There are known to be age-related changes in metabolite concentrations and relaxation times, e.g. tCho, tCr and mI increase modestly with age [38–42]. In addition, small vessel disease is associated with reduced tNAA levels in white matter [43, 44]. Furthermore, cerebral atrophy and neurological comorbidities such as Parkinson’s disease and dementia are prevalent in this population, and may confound through altering baseline metabolite levels [45–55]. Hence, we performed exploratory analyses for age, atrophy, or neurological comorbidity-related variations in metabolite levels.

### Data availability

The data derived from this study are available from the corresponding author, upon reasonable request.

## Results

Thirty-eight participants were recruited, all inpatients due to an emergency medical admission. Thirteen participants (nine delirium, four no delirium) were unable to complete the full MRS protocol due to inability to tolerate MR scan (*n* = 5); scanner unavailability (*n* = 2); deterioration in clinical condition (*n* = 3); discharge from hospital (*n* = 2) or withdrawal from participation due to change in clinical circumstance (*n* = 1). Thirteen participants with delirium (eight men, five women) and 12 without (‘no delirium’) (five men, seven women) completed the MRS protocol. Across the delirium cohort, a mix of hypoactive, mixed and hyperactive signs and symptoms of delirium were seen. From these there were nine delirium and 11 no delirium patients in whom DWI data was acquired that was suitable for generating ADC maps.

Among the cohort that underwent MRS, seven patients had known neurodegenerative co-morbidities: five with dementia (delirium *n* = 3; no delirium *n* = 2), one with Parkinson’s disease and one with progressive supranuclear palsy (PSP) (both in the delirium group). The commonest reasons for hospital admission were infection (*n* = 12), fall (*n* = 5), one each of acute coronary syndrome, acute kidney injury, epistaxis, lacunar stroke, Colles’ fracture, hypothyroidism, hyponatraemia and delirium with unspecified cause. All spectra exceeded the recommended quality thresholds of SNR and FWHM [34].

### Group comparisons

There was no significant sex (Chi = 0.99, *P* = .32) or age difference between groups (Table 1A). As expected, significant differences between delirium and no delirium patients were seen in MDAS (15 vs 2.5; *P* < .001) and OSLA (7 vs 0; *P* < .001), but also in CFS (5 vs 4.5; *P* = .049). IQCODE and APACHE II scores were not significantly different between groups. Ten delirium but only three no delirium patients had an IQCODE score predictive of dementia in a delirium cohort (>3.82) [56].

**Table 1:** Summary of Clinical (A) and MRI parameters (B) and group comparison.

1A Clinical parameters					
		Delirium	No delirium	Significance (p)	Test
Proportion of group completing MRS acquisition	N	13 (57%)	12 (75%)		
Gender (M:F)	N	8:5	5:7	0.040	X^2^
Age	Mean Range	82 67–89	79 67–90	0.152	MW
Relevant past medical history	N				
	Dementia PD/PSP Stroke	3 2 0	2 0 2		
Clinical assessments	Median and range				
	MDAS Total	15 8–26	2.5 0–9	<0.001	MW
	OSLA	7 4–12	0 3–13	<0.001	MW
	APACHE-II	8 6–21	6.5 5–10	0.068	MW
	CFS	5 4–8	4.5 1–7	0.049	MW
	IQCODE	4 0–5	3 1.43–4.43	0.095	MW
1B MRI parameters (mean and standard deviation)					
	Delirium	No delirium	Significance (p)	Test	
Participants (N)	13	12			
FWHM (ppm)	0.04 (0.01)	0.05 (0.01)	0.137	MW	
SNR	16.1 (4.2)	13.5 (4.9)	0.205	MW	
Fazekas score	1.8 (0.7)	2.2 (0.8)	0.354	KS	
GCA scale	17.2 (7.0)	21.5 (4.5)	0.200	KS	
Median ADC (*N* = 9: 11)^a^	0.97 (0.17)	0.86 (0.09)	0.552	MW	

X^2^ = Chi Squared

MW = Mann–Whitney U

KS = Kolmogorov Smirnov

^a^ADC map data not available for all patients due to data quality and early termination of MRI scan

Measures of MRS quality (FWHM and SNR), and Fazekas and atrophy scores, were not significantly different between groups (Table 1B). The median ADC within the MRS voxel was not significantly different between groups. For both groups combined there was a significant increase in median ADC with Fazekas score (ISJT test *P* = .002) indicating the white matter damage within the MRS voxel paralleled the overall whole brain score of white matter damage.

**Figure 2:**
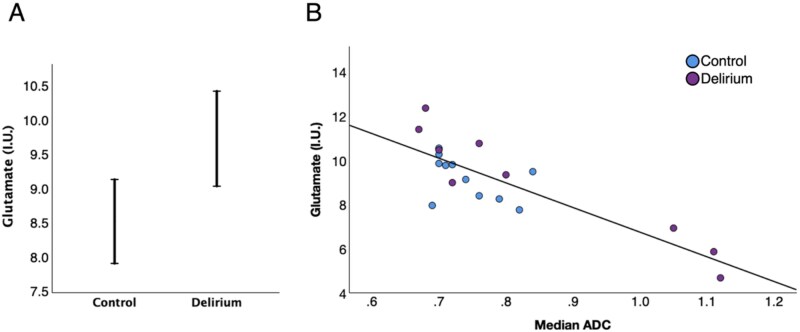
Evidence of elevated Glu in delirium patients. (A) From our GLM analysis the marginal mean of Glu is 15% higher in delirium patients compared to controls across the cohort. (B) Linear regression of estimated Glu with the tissue ADC. When the tissue ADC is ≈0.68 mm^2^/s, a rate equivalent with [74], there is an 11% increase in Glu in delirium patients compared to controls.

General Linear Model (GLM) analysis revealed that most metabolites (Glu, tNAA, tCr and Glx) showed a strong and significant inverse correlation with median ADC as might be expected (Table 2), due to cell density reductions and increased reference water signal that occurs with oedema. Only Glu was significantly different between groups (*P* = .024) as shown by the plots of the marginal means from the GLM analysis (Figure 2A) and correlation plot (Figure 2B). At the lowest ADC of ≈0.68 mm^2^/s, which would be associated with NAWM, we estimate there is an 11% increase of Glu in delirium compared to controls, and on average a 15% increase (Figure 2A).

**Table 2:** GLM analysis results for each metabolite as the dependent variable, showing the *P*-values for their dependence on group (delirium participants or controls) age, median ADC, FWHM and SNR.

	Gln	Glu	Ins	tCho	NAA	tCr	Glx
Group (delirium vs control)	0.860	0.024	0.948	0.587	0.096	0.947	0.046
Age	0.273	0.135	0.032	0.393	0.514	0.804	0.058
Median ADC	0.441	<0.001	0.203	0.086	<0.001	0.004	<0.001
FWHM	0.407	0.091	0.998	0.929	0.623	0.239	0.059
SNR	0.681	0.219	0.711	0.294	0.004	0.102	0.453

To eliminate the possibility that this effect was due to neurological comorbidity, we performed an analysis only in patients without neurological comorbidities (*n* = 5 delirium, *n* = 9 no delirium). Glu was still significantly higher (*P* = .037) in the delirium group, but there were too few data for the full GLM to assess those with neurological comorbidities alone (*n* = 4 delirium, *n* = 2 no delirium). We did not find that Glu was different between groups with and without neurological comorbidities (*P* = .593) for a GLM with neurological comorbidities as a factor instead of group. Overall, the data suggests the Glu elevation is related to delirium and not to other pathological effects.

### Age and atrophy

**Figure 3:**
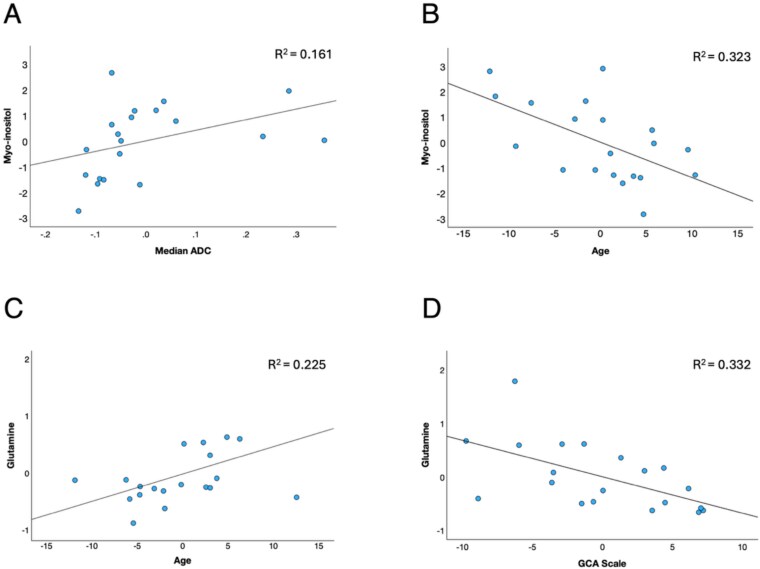
Regression analysis of delirium and control patient data combined, for mI with median ADC and age as covariates, and for Gln with median ADC with age and GCA scale as covariates. (A) Positive correlation of mI with median ADC. (B) Negative correlation of mI with age. (C) Positive correlation of Gln with age. (D) Negative correlation of Gln with GCA scale.

The GLM analysis indicates no group difference in mI, but there was an effect of age (*P* = .032). In a simple regression analysis across both groups mI showed a positive correlation with median ADC and negative correlation with age (Figure 3A and B). In both delirium and no delirium patients there was a significant reduction in mI from age 67 to 90 (R = –0.556, *P* = .004). These data combined suggest an intracellular increase in mI related to white matter damage, and an age-related decrease. When including GCA score as a covariate in our GLM of metabolite levels we found that Gln as the dependent variable was significantly correlated with GCA scale (*P* = .028), with a near-significant age effect (*P* = .057). There was still a significant elevation of Glu in delirium patients (*P* = .046) and a significant correlation of mI with age (*P* = .039), albeit at slightly reduced p-value due to the extra covariate. Regression analysis of Gln with median ADC showed age (*P* = .047) and GCA scale (*P* = .024) (Figure 3C and D) as significant covariates.

## Discussion

In this study we examined brain metabolite concentrations during delirium in a typical cohort of older hospitalised patients. We found on average 15% greater levels of Glu in delirium patients compared to age and physical illness severity-matched patients without delirium. In both patient groups combined, mI increased with white matter damage and decreased with age, whereas Gln increased with age and decreased with GCA.

Our findings are consistent with animal models of delirium, in which cerebral Glu (or Glx) is increased and correlates with acute cognitive deficits [18–20].

Glx has been shown to correlate with dementia risk score in mid-life, suggesting that in people without established neurodegenerative disease, higher levels might indicate cerebral vulnerability [57]. Raised CSF Glu concentration is predictive of delirium following surgery for hip fracture [21]. In contrast, MRS has generally shown reduced Glu or Glx concentrations in Parkinson’s disease [47], Parkinson’s disease dementia [48], dementia with Lewy bodies [49, 58], Alzheimer’s disease [50–53], as well as healthy older adults [59]. This suggests that the increase in cerebral Glu concentration in our delirium group is not driven by greater prevalence of background neuropathology compared to the control group.

How might increase cerebral Glu correlate with delirium symptoms and/or make the brain more vulnerable to subsequent cognitive decline? Astrocytes are central to Glu metabolism; when activated by inflammatory signals from the periphery they take up less Glu [60]. In delirium, acute alterations in astrocyte biology could result in reduced ability to bind Glu through downregulation of Glu receptors such as Glu transporter 1 (GLT-1) and Glu/aspartate transporter (GLAST) [61]. This could lead to an increase in extracellular Glu concentration with consequent excitatory neurotoxicity. This has been demonstrated in pre-clinical models, in which delirium causes increased astrocyte reactivity, raised Glu concentration, synaptic injury in the hippocampus and neurocognitive deficits [19, 62]. Glu-mediated damage to relevant brain regions such as the hippocampus incurred during delirium might reduce the ability of the brain to withstand an established level of neuropathology, e.g. through impaired long-term potentiation [63] resulting in earlier clinical expression of dementia.

Ours is not the first report of raised cerebral Gln concentrations with ageing; a meta-analysis of MRS studies showed a positive correlation between cerebral Gln levels and age [59]. Aged mouse astrocytes undergo upregulation of GLT-1, GLAST and Gln synthetase (GS), and older human hippocampi show increased numbers of GS positive cells and GS and GLT-1 levels [64]. Such changes would be expected to increase astrocyte Gln levels, suggesting a shift in cerebral Glu–Gln cycle towards Gln in the healthy older brain that could represent a neuroprotective mechanism against glutamatergic excitotoxicity [64].

The decline in Gln concentration with brain atrophy likely reflects loss or dysfunction of astrocytes, the main cell type in which glutamine synthesis occurs. This contrasts with our finding of increased Gln with ageing and suggests that normal ageing alone produces different effects in the brain to the combination of pathological processes that underlie cerebral atrophy.

In healthy ageing tCr, tCho and mI increase from age 20 to 60 [40]. We observed a significant reduction in mI from age 67 to 90 in both delirium and no delirium patients (R = -0.556, *P* = .004). mI is an osmolyte elevated in inflammatory diseases [65, 66] and mild cognitive impairment [67] suggesting a contribution to progression of neurodegenerative processes. The reduction in mI with age that we observed may represent age-related glial dysfunction [68] independent of whether patients have delirium. It could also reflect acute medical illness which was a defining feature of our cohort. Further studies of metabolite levels in non-hospitalised older people with varying levels of brain atrophy are needed to help understand these metabolite changes.

To our knowledge, this is the first report of MRS in a typical older delirium population. A previous study used MRS to study delirium in a younger cohort of patients undergoing BMT for haematological malignancy [*n* = 5 BMT patients with delirium (mean age 63.8), nine BMT patients without delirium, nine healthy controls] [22]. A single large volume of interest, consisting of white matter from frontal, parietal and temporal lobes was used for multivoxel MRS acquisition from which global averages of metabolite ratios were reported. In contrast to our findings, the authors did not find any between-group differences in the relative concentration of Glx.

The study was performed in a significantly younger cohort in whom delirium might be associated with different biochemical effects in the brain than in the typical older delirium population. Other investigators have also found elevated plasma Glu levels in frailty [69, 70] but to our knowledge, no other data on brain Glu concentrations in acutely unwell frail older adults have been published.

Taken together our data and that from previous studies suggest that ageing, cerebrovascular disease and neurodegeneration produce (sometimes contradictory) changes that potentially confound the detection of metabolic differences in delirium, a condition characterised by advanced age and a high prevalence of neurological comorbidities.

Our study has several strengths. We recruited participants with the typical profile of the older frail adult with delirium. This group was compared with a ‘no delirium’ group, matched for age, physical illness severity and setting.

Limitations of our study include delirium presence and severity being assessed on only one occasion. It is possible that delirium might have resolved, or developed in ‘no delirium’ patients, in the time period between the clinical assessment and MRI scan. However, we assume that biochemical changes in the brain associated with delirium do not resolve immediately. We did not record whether patients were on medications known to affect Glu concentrations such as riluzole [71] or memantine [72] however, we had no patients with amyotrophic lateral sclerosis, whilst memantine was not in widespread use for Alzheimer’s disease in the UK at the time of this study. Due to our small sample size, we may not have captured the extent of individual level differences in cognitive assessment or MRI characteristics which exist within patients with and without delirium. Finally, our delirium and no delirium groups were not completely matched for pre-admission frailty. This was not unexpected. Older hospitalised patients with delirium are significantly less independent in activities of daily living, less well-nourished and frailer than their non-delirious counterparts [73].

## Conclusion

This study demonstrates that MRS can effectively investigate neurochemical changes in frail older patients with delirium. Our observations of elevated cerebral Glu in older adults with delirium and of mI increasing with white matter damage and reducing with age require confirmation. However, Glu mediated excitotoxicity is a potentially modifiable contributor to both the acute delirium syndrome and post-delirium cognitive decline that merits further investigation.

## Supplementary Material

Supplementary_materials_afag013

## Data Availability

The data derived from this study are available from the corresponding author, upon reasonable request.
