# Supplementary material for: Magnetic resonance spectroscopy in hospitalised older people shows age and delirium-specific metabolic changes
Source: Age Ageing. 2026 Feb 1;55(2):afag013. doi: 10.1093/ageing/afag013 (PMC12862978; doi:10.1093/ageing/afag013)
Supplement: Supplementary_materials_afag013 [file supplementary_materials_afag013.docx]

**Magnetic Resonance Spectroscopy in Hospitalised Older People Shows Age and Delirium-Specific Metabolic Changes**

**Appendix 1. Acquisition and processing details for MRI and MRS**

Structural brain imaging and quantitative 1H MRS were acquired using a 3-Tesla Achieva dual TX magnetic resonance scanner (Philips Medical Systems, Eindhoven, The Netherlands) equipped with a 32-channel head coil. The total acquisition time for both MRI and MRS was 22 minutes.

***MRI***

Image acquisition included the following sequences to evaluate brain structure and identify confounding neuropathology: High resolution 3D T1-weighted (T1w) (sagittal with 1.12 mm isotropic voxels); 3D Fluid Attenuated Inversion Recovery (FLAIR), (sagittal 1.12mm x 0.56mm x reformatted to 5mm thick axial for Fazekas scoring); diffusion weighted imaging (DWI), (axial with 0.9mm x 0.9mm x 5mm spatial resolution, and b=0 & 1000 s mm^-2^); susceptibility weighted imaging (SWI) (0.5mm isotropic).

Apparent diffusion coefficient (ADC) maps were created from the DWI assuming the standard exponential decay function for the two images acquired with diffusion weightings of b=0 and b=1000 s mm^-2^.

***MRS***

1H MRS was acquired from a voxel placed within parietal white matter centred on the centrum semiovale using PRESS localisation, with TR 2000ms, TE 32ms, 128 averages of water suppressed metabolite signals, and 8 averages of water signal. T1w images were used for anatomical placement and FLAIR images used to minimise contributions from White Matter Hyperintensities (WMH, which represent areas of small vessel disease associated with ageing, hypertension, and other vascular risk factors). Voxel dimensions were chosen to maximise the volume of white matter, whilst excluding signal contributions from cerebrospinal fluid and grey matter, and ranged from 15mm to 20mm in each dimension (20mm cubic voxel shown in Figure 1 main text), with smaller voxels used for patients with greater atrophy.

Metabolite quantitation was with LCModel using the tissue water signal as a reference, assumed 41.7M, and no corrections made for T1 or T2 relaxation time effects, hence although nominally in mM are presented as institutional units (IU).

***MRS analysis***

Definition and selection of good quality spectra and initial MRS data analysis was made by an experienced MRI physicist (FAH) who was blinded to patient group. Prior to group analysis, spectra were excluded if visual assessment indicated unacceptable artefacts, or if water signal linewidth Full-width at Half Maximum (FWHM) was > 0.1ppm, or metabolite signal-to-noise ratio (as defined by LCModel) was < 3. Ultimately, no spectra were rejected due to issues of poor water suppression, low signal-to-noise, baseline roll or artefacts that might be associated with patient movement, and all exceeded the recommended quality criteria.

Since metabolite concentrations are calculated with reference to the tissue water signal, the presence of oedema from white matter damage will be a confounding factor due to changes in tissue water content and cell density, which also alters the tissue relaxation times. Although many studies use metabolite ratios, for which the effects of water content and relaxation time variations will cancel out, it is not known which, if any, metabolite could be considered to provide a stable reference signal. Hence for comparisons of metabolites between delirium and no delirium patient groups we chose a General Linear Model (GLM) approach.

The apparent diffusion coefficient (ADC) is sensitive to water content and cell density, hence median ADC within the MRS voxel was used as a covariate in a GLM to assess group metabolite differences in the presence of such variations. Median ADC was chosen in preference to the mean as some voxels included mixed tissue of normal appearing white matter (NAWM) and WMH and create a bi-modal distribution of ADCs. In-house software was used to map the MRS voxel onto the ADC maps to calculate the median ADC within the spectroscopy voxel.
